# AI-CAD-Guided Mammographic Assessment of Tumor Size and T Stage: Concordance with MRI for Clinical Staging in Breast Cancer Patients Considered for NAC

**DOI:** 10.3390/tomography11070072

**Published:** 2025-06-24

**Authors:** Ga Eun Park, Kabsoo Shin, Han Song Mun, Bong Joo Kang

**Affiliations:** 1 Department of Radiology, Seoul St. Mary’s Hospital, College of Medicine, The Catholic University of Korea, Seoul 06591, Republic of Korea; hoonhoony@naver.com (G.E.P.); lionmain@catholic.ac.kr (B.J.K.); 2 Division of Medical Oncology, Department of Internal Medicine, Seoul St. Mary’s Hospital, College of Medicine, The Catholic University of Korea, Seoul 06591, Republic of Korea; kabsoo.shin@catholic.ac.kr

**Keywords:** breast neoplasms, neoadjuvant therapy, artificial intelligence, mammography, neoplasm staging

## Abstract

**Objectives**: To evaluate the agreement between AI-CAD-guided mammographic and MRI measurements of tumor size and T stage in breast cancer patients being considered for neoadjuvant chemotherapy (NAC). **Methods**: This retrospective study included 144 women (mean age, 52 ± 11 years) with invasive breast cancer who subsequently received NAC and underwent both AI-CAD mammography (score ≥ 10) and pre-treatment MRI. Tumor sizes from AI-CAD contours were compared with MRI using Pearson correlation, intraclass correlation coefficients (ICCs), and Bland–Altman analysis. Concordance was defined as a ±0.5 cm difference. The contour showing the highest agreement was used to compare T stage with MRI using weighted kappa. **Results**: The mean AI-CAD abnormality score was 86.3 ± 22.2. Tumor sizes on mammography were 3.0 ± 1.2 cm (inner), 3.8 ± 1.5 cm (middle), and 4.8 ± 2.2 cm (outer), while the MRI-measured tumor size was 4.0 ± 1.9 cm. The middle contour showed the strongest correlation with MRI (r = 0.897; ICC = 0.866), the smallest mean difference (–0.19 cm; limits of agreement, –1.87 to 1.49), and the highest concordance (61.1%). Agreement was higher in mass-only lesions than in NME-involved lesions (ICC = 0.883 vs. 0.775; concordance, 70.9% vs. 46.6%). T stage comparison using the middle contour showed substantial agreement with MRI (κ = 0.743 [95% CI, 0.634–0.852]; agreement, 88.2%), with higher concordance in mass-only lesions (93.0%) than NME-involved lesions (81.0%) and more frequent understaging in the latter (17.2% vs. 2.3%). **Conclusions**: AI-CAD-guided mammographic assessment using the middle contour demonstrated good agreement with MRI for tumor size and T stage, indicating its value as a supportive tool for clinical staging in MRI-limited settings.

## 1. Introduction

Breast cancer is among the most prevalent malignancies in women, accounting for nearly 12% of all cancer cases globally [1,2]. Accurate assessment of tumor size and extent is a key factor in breast cancer evaluation, guiding therapeutic decisions and offering prognostic insight into overall survival [3,4]. This assessment also plays a significant role in determining whether patients should undergo upfront surgery or receive neoadjuvant chemotherapy (NAC) prior to surgery.

NAC is commonly indicated for patients with locally advanced breast cancer (T2 or greater, node-positive disease), as well as for those with triple-negative or HER2-positive subtypes, or when downstaging is necessary to enable breast-conserving surgery [5]. Inflammatory breast cancer and cases where the extent of residual disease may influence subsequent treatment decisions are also appropriate candidates for NAC [6]. By reducing tumor burden before surgery, NAC facilitates surgical planning and provides important prognostic information [7,8].

In this context, breast magnetic resonance imaging (MRI) is widely regarded as the most accurate imaging modality for evaluating tumor extent and treatment response, and is, therefore, a reasonable comparative modality for evaluating other imaging approaches [9,10,11]. MRI findings often play a decisive role in determining whether patients should be considered for NAC, especially when assessing tumor size and clinical T stage.

Mammography, a fundamental imaging modality for breast cancer diagnosis, has long served as the standard screening tool [12]. The incorporation of artificial intelligence-based computer-aided detection (AI-CAD) into mammographic interpretation has led to significant advancements. AI-CAD has been shown to improve cancer detection rates by 0.7–1.6 per 1000 screened women and increase the positive predictive value (PPV) by 0.1–1.9% when used as a supplementary reader, with minimal increases in recall rates (0.16–0.30%) [13]. Large multi-center studies have demonstrated that commercially available AI-CAD systems achieve comparable or even superior sensitivity and specificity compared to single or double readings by radiologists [14,15]. Moreover, AI-CAD enhances reading efficiency, reduces inter-reader variability, and decreases radiologists’ workload. In dual reading settings, AI-CAD has shown performance sufficient to replace one reader, with additional readings needed in only 4–6% of cases [13,15,16].

Although AI-CAD systems have been extensively evaluated for cancer detection in screening settings, few studies have explored their diagnostic potential beyond detection—particularly in the context of tumor size estimation or clinical staging. To our knowledge, no prior study has quantitatively examined the agreement between AI-CAD-based lesion size on mammography and MRI measurements in breast cancer patients being considered for NAC. Given the clinical importance of tumor size in staging and treatment planning, along with the widespread availability of mammography even in resource-limited settings, such an investigation may support broader diagnostic applications of AI-CAD and offer supportive information in situations where MRI is unavailable or limited.

Therefore, the aim of this study was to evaluate the agreement between AI-CAD-guided mammographic and MRI measurements of tumor size and clinical T stage, in order to determine whether AI-CAD mammography can provide supportive information for clinical staging in breast cancer patients being considered for NAC.

## 2. Materials and Methods

### 2.1. Study Population and Clinical Data Collection

**Figure 1:**
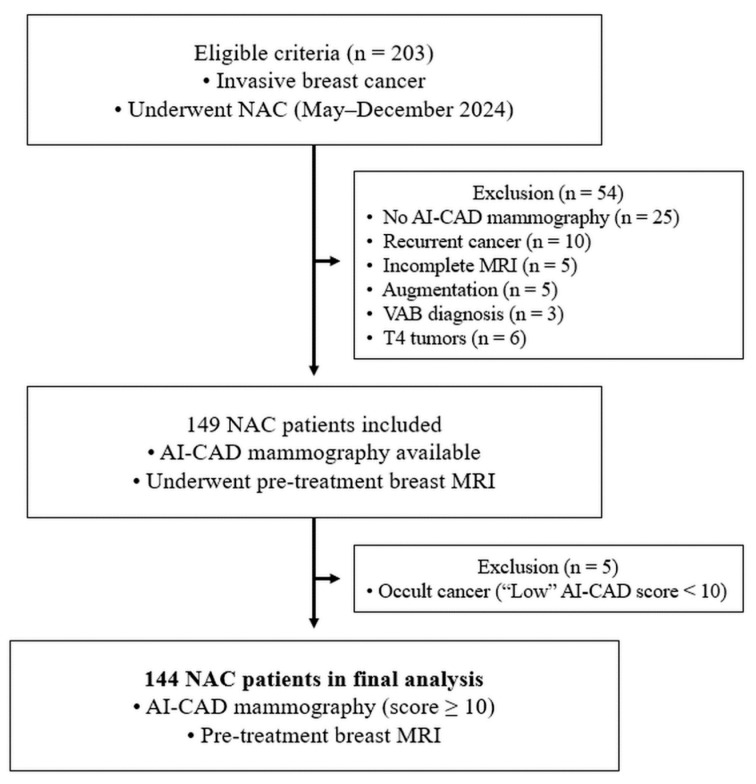
Patient selection flowchart for an AI-based mammography study. NAC = neoadjuvant chemotherapy; AI-CAD = artificial intelligence-based computer-aided detection; VAB = vacuum-assisted biopsy.

We identified 203 consecutive patients diagnosed with invasive breast cancer who subsequently underwent NAC between May and December 2024 at our tertiary referral center. Among them, 54 patients were excluded for the following reasons: mammography not processed with AI-CAD or AI-CAD output was unavailable (*n* = 25), treatment for recurrent breast cancer (*n* = 10), incomplete or suboptimal pre-treatment breast MRI (*n* = 5), augmentation mammoplasty for cosmetic purposes (*n* = 5), diagnosis based on vacuum-assisted biopsy (*n* = 3), and T4 tumors (*n* = 6), which were excluded because their classification is based on chest wall or skin invasion rather than tumor size. After exclusions, 149 patients with both pre-treatment mammography and breast MRI were initially included. Of these, 5 patients with occult breast cancer—defined as an AI-CAD abnormality score < 10 (which was presented as “low”) and interpreted as a test-negative result—were further excluded. Consequently, a total of 144 patients (mean age, 52 ± 11 years) were included in the final analysis to evaluate the agreement between AI-CAD-guided mammographic and MRI-based tumor size and T stage assessments, which were performed prior to NAC initiation and used to inform clinical staging (Figure 1).

Clinical data were retrieved from electronic medical records and included age, menopausal status (premenopausal or postmenopausal), family history of cancer (none, breast cancer, or other cancer), and BRCA1/2 mutation status (yes, no, or unknown [not tested]).

### 2.2. Pathologic and Biomarker Assessment

The histologic type (invasive ductal, invasive lobular, or others), histologic grade (I–II or III), hormone receptor (HR) status—including estrogen receptor (ER) and progesterone receptor (PR)—HER2 expression, and Ki-67 expression were determined from the initial pathology reports of core biopsies obtained before NAC. HR status was defined as positive if tumor cells showed ER and/or PR expression, with a cut-off of an Allred score ≥ 3 [17]. HER2 positivity was defined as an immunohistochemistry (IHC) score of 3+ or 2+ with a positive silver fluorescence in situ hybridization (SISH) result, in accordance with the 2018 ASCO/CAP guidelines [18]. Ki-67 expression was categorized as high (≥20%) or low (<20%) based on the 2013 St. Gallen consensus [19].

Based on ER, PR, and HER2 status, tumors were further classified into molecular subtypes: HR+/HER2–, HR+/HER2+, HR–/HER2+, and HR–/HER2– (triple-negative breast cancer, TNBC).

### 2.3. MRI Acquisition and Interpretation

Breast MRI was performed using 3T scanners (Verio and Vida, Siemens Healthcare, Erlangen, Germany) with patients in a prone position using a dedicated breast coil. The protocol included axial T2-weighted imaging, diffusion-weighted imaging (DWI), and T1-weighted dynamic contrast-enhanced (DCE) imaging. DCE-MRI was obtained before and after intravenous injection of a gadolinium-based contrast agent (Gadovist, 0.1 mmol/kg). The Verio scanner acquired images at 10, 70, 130, 190, 250, and 310 s using a 3D volumetric interpolated breath-hold examination (VIBE) sequence, while the Vida scanner acquired images at 10, 93, 176, 259, 342, and 425 s using a 3D fast low-angle shot (FLASH) sequence. All DCE sequences were acquired with fat suppression. DICOM data from DCE-MRI were processed using syngo.via VB80 (Siemens Healthcare, Erlangen, Germany) to generate maximum intensity projection (MIP) images and subtracted images for all post-contrast phases.

An experienced breast radiologist (B.J.K., with 19 years of experience) independently reviewed all pre-treatment breast MRI scans in a blinded manner. The radiologist was informed only of the laterality (right or left) of the breast cancer but were blinded to detailed pathological findings, including histologic subtype, biomarker status, and the use of neoadjuvant chemotherapy. Background parenchymal enhancement (BPE) was first assessed and categorized as minimal, mild, moderate, or marked. Lesions were then classified based on their morphological appearance into one of the following categories: mass, non-mass enhancement (NME), or mass with NME. For each case, tumor size assessment was performed with reference to three MRI sequences: axial T2-weighted imaging, early-phase subtracted T1-weighted DCE imaging, and MIP images [20]. The greatest tumor dimension among the three sequences was measured using electronic calipers. When multiple lesions were present, the most suspicious lesion was used for size measurement. Based on the recorded size, MRI-based T staging was subsequently determined by a medical oncologist (K.S.).

### 2.4. AI-Based Mammographic Analysis

Bilateral mammograms were obtained using a dedicated digital mammography system (Selenia Dimensions; Hologic, Marlborough, MA, USA), including standard craniocaudal (CC) and mediolateral oblique (MLO) views. All images were analyzed using a commercially available AI-CAD program (Lunit INSIGHT MMG; https://insight.lunit.io, version 1.1.8.2, accessed on 23 June 2025), which has been validated in multiple international studies [21,22,23,24,25]. The system provides breast parenchymal composition (categorized as A to D: fatty, scattered, heterogeneously dense, or extremely dense) and a corresponding breast density score (0–10) for all cases. It also provides abnormality scores (0–100) for each breast, along with grayscale contour maps and the type of abnormality (mass, calcification, or mass with calcification), if present. An abnormality score ≥ 10 was considered positive, and patients with scores < 10 (n = 5) were excluded as described in the inclusion criteria.

Lunit INSIGHT MMG (Lunit, Seoul, Republic of Korea) is an AI-CAD software for breast cancer detection in mammography, developed using deep convolutional neural networks (CNNs) [23]. It employs ResNet-34, a widely used CNN architecture, as the backbone network [26]. The algorithm was trained on more than 200,000 mammographic cases collected from Korea, the United States, and the United Kingdom, and has received regulatory approval from the Korean Ministry of Food and Drug Safety.

For each case, the lesion with the highest abnormality score was selected from the more suspicious side (in bilateral cases, n = 4) and from the projection view (CC or MLO) with the higher score. Two board-certified breast radiologists (G.E.P. and H.S.M., with 9 and 14 years of experience, respectively), who were blinded to MRI findings, evaluated lesion size by consensus based on AI-generated contours. When a lesion was detected, the AI-CAD system generated up to three concentric contours according to abnormality score thresholds: inner (≥90), middle (50–89), and outer (10–49). Each contour represented a distinct level of algorithm-predicted probability of malignancy. Tumor size was measured along the longest axis between the edges of each contour line, excluding line thickness to avoid overestimation. When fewer than three contours were present, the following approach was applied: if only one contour was present, the same value was assigned to all three contours; if two contours were present, the middle value was interpolated as the average of the inner and outer contours. Pixel spacing (0.1670 mm/pixel) was validated in 20 randomly selected cases and was used to convert all sizes measured by radiologists using AI-CAD contours from pixels to centimeters. Tumor sizes derived from each contour were used to assign three separate mammography-based T stages per case (K.S.). Tumor laterality was shared with MRI reviewers to ensure consistency in lesion matching across modalities. As a result, a total of 144 representative lesions from 144 patients were included in the final analysis.

### 2.5. Statistical Analysis

Descriptive statistics were used to summarize clinicopathologic and imaging characteristics. Continuous variables were reported as mean ± standard deviation, and categorical variables as counts and percentages.

Pearson correlation coefficients were calculated to assess linear agreement between AI-CAD-guided and MRI tumor sizes. Intraclass correlation coefficients (ICCs) were computed using a two-way random effects model (ICC[2,1]) to evaluate absolute agreement. ICC values were interpreted as follows: <0.50, poor; 0.50–0.75, moderate; 0.75–0.90, good; >0.90, excellent [27]. Bland–Altman analysis was also performed to assess mean differences and 95% limits of agreement (LoA). Concordance between tumor sizes obtained from each modality was defined as a difference within ±0.5 cm, reflecting a clinically acceptable threshold that is widely used in breast imaging and supported by prior studies [20,28,29,30].

For T staging, AI-CAD-guided stages derived from different contour levels were compared with MRI-based stages. The best-performing contour was used for the final comparison. Quadratic weighted kappa statistics, appropriate for ordinal data, were used to assess agreement, with interpretation as follows: <0.00, poor; 0.00–0.20, slight; 0.21–0.40, fair; 0.41–0.60, moderate; 0.61–0.80, substantial; 0.81–1.00, almost perfect [31]. Rates of agreement, understaging (AI-CAD-guided T stage < MRI T stage), and overestaging (AI-CAD-guided T stage > MRI T stage) were also calculated.

All statistical analyses were performed using SPSS (version 28.0; IBM Corp., Armonk, NY, USA), with significance set at *p* < 0.05.

## 3. Results

### 3.1. Baseline Clinicopathologic and MRI Characteristics

A total of 144 representative lesions from 144 patients (mean age, 51.7 ± 10.8 years) were included in the final analysis (Table 1). Of these, 86.1% were aged 40 years or older. A family history of breast cancer was reported in 13.9% of patients, and BRCA1/2 mutations were confirmed in 4.2%. 

Most tumors were invasive ductal carcinoma (93.1%), while invasive lobular and other histologic types each accounted for 3.5%. High-grade tumors (grade III) were observed in 43.8% of cases. ER and PR positivity were identified in 56.2% and 39.6% of patients, respectively, and HER2 positivity was observed in 45.1% of cases based on IHC/SISH. According to HR and HER2 status, the most common molecular subtype was HR–/HER2– (TNBC, 30.6%), followed by HR–/HER2+ (29.9%), HR+/HER2– (24.3%), and HR+/HER2+ (15.3%). High Ki-67 expression (≥20%) was found in 84.0% of tumors.

Regarding MRI characteristics, minimal and mild BPE were the most frequently observed patterns (40.3% and 35.4%, respectively). In terms of lesion type, mass lesions only were present in 59.7% of patients, mass with NME in 30.6%, and NME only in 9.7%. The mean tumor size on MRI was 4.0 ± 1.9 cm (range, 1.7–10.8 cm). Based on the MRI-derived T stage, T2 was the most prevalent (100/144, 69.4%), followed by T3 (35/144, 24.3%) and T1 (9/144, 6.2%).

### 3.2. AI-CAD-Guided Mammographic Tumor Assessment

Among the 144 cases, the most common breast composition was heterogeneously dense (66.0%), followed by scattered (18.1%) and extremely dense (15.3%). The mean breast density score (0–10) was 6.9 ± 1.6 (Table 2).

**Figure 2:**
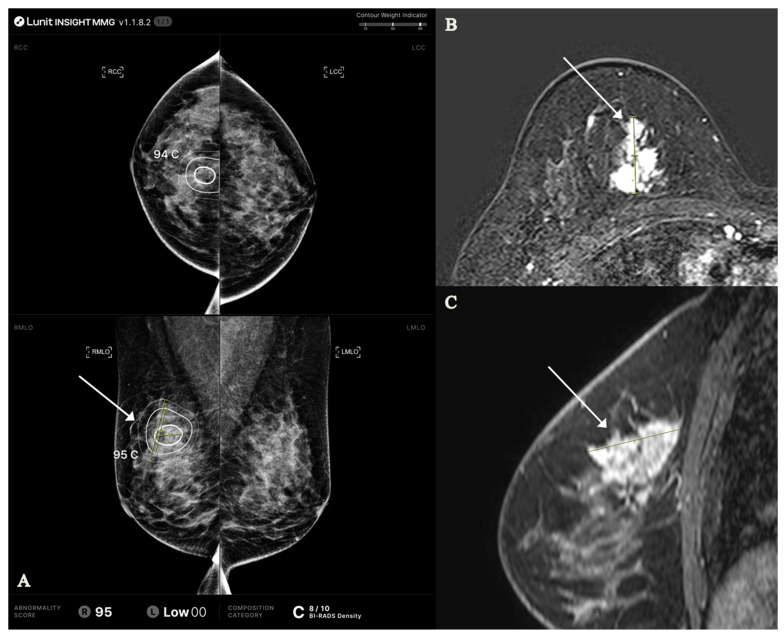
AI-CAD mammography and MRI findings in a 48-year-old woman with invasive ductal carcinoma, grade II (HR+/HER2+, Ki-67 index 34%). (**A**) AI-CAD mammography shows heterogeneously dense breasts (category C, density score 8/10) and a high abnormality score (95) on the right mediolateral oblique view. The lesion was classified as calcification. Tumor size along the inner, middle, and outer contours was 2.0, 3.5, and 4.9 cm, respectively (T1, T2, T2). (**B**,**C**) Contrast-enhanced MRI reveals a heterogeneously enhancing mass with non-mass enhancement in the corresponding area. The lesion measured 3.3 cm on axial and 3.7 cm on sagittal views; the maximal diameter (3.7 cm) was used for T2 staging. Arrows indicate the lesion; yellow lines represent caliper-based measurements. AI-CAD = artificial intelligence-based computer-aided detection; HR = hormone receptor; HER2 = human epidermal growth factor receptor 2.

For the AI-CAD-detected lesion, the mean abnormality score (0–100) was 86.3 ± 22.2 (Figure 2). The most frequently detected abnormality type was mass with calcification (43.8%), followed by mass (40.3%) and calcification alone (16.0%). The MLO view was more frequently selected for analysis (59.7%) compared to the CC view (40.3%). In terms of the number of abnormality contours, three contours were identified in the majority of cases (68.1%).

The mean tumor sizes derived from the inner, middle, and outer contours were 3.0 ± 1.2 cm, 3.8 ± 1.5 cm, and 4.8 ± 2.2 cm, respectively. Based on AI-CAD-guided mammographic T staging, T2 was the most frequent stage across all three contours (inner: 69.4%; middle: 79.9%; outer: 66.0%). The proportion of T3 classification increased with contour size, whereas T1 classification was more common in the inner contour than in the middle or outer contours.

### 3.3. Agreement Between MRI and AI-CAD-Guided Mammographic Tumor Size Measurements

**Figure 3:**
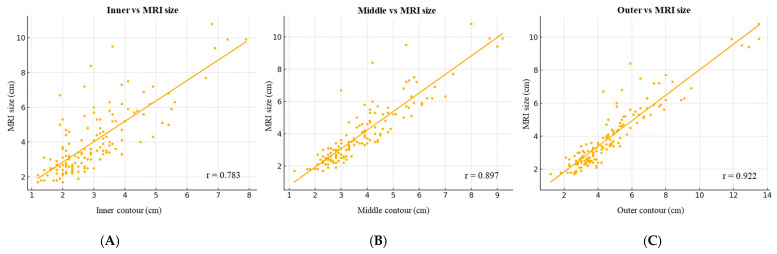
Scatter plots showing Pearson correlations between MRI and AI-CAD-guided tumor measurements based on (**A**) inner, (**B**) middle, and (**C**) outer contours (r = 0.783, 0.897, and 0.922; all *p* < 0.001). AI-CAD = artificial intelligence-based computer-aided detection.

Pearson correlation analysis revealed strong linear associations between MRI-measured tumor size and AI-CAD-guided measurements from all contour levels (inner: r = 0.783, *p* < 0.001; middle: r = 0.897, *p* < 0.001; outer: r = 0.922, *p* < 0.001) (Figure 3).

ICC analysis demonstrated good agreement for the middle and outer contours (ICC = 0.866 [95% CI, 0.815–0.902] and 0.847 [95% CI, 0.446–0.936], respectively), whereas the inner contour showed only moderate agreement (ICC = 0.602 [95% CI, 0.153–0.794]) (Table 3). When analyzed by MRI lesion type, ICCs were consistently higher in mass-only lesions compared to NME-involved lesions, which included both NME-only lesions and masses with associated non-mass enhancement. The highest agreement was observed in mass-only lesions using the middle contour (ICC = 0.883 [95% CI, 0.827–0.922]), whereas the lowest was found in NME-involved lesions using the inner contour (ICC = 0.443 [95% CI, 0.000–0.729]).

**Figure 4:**
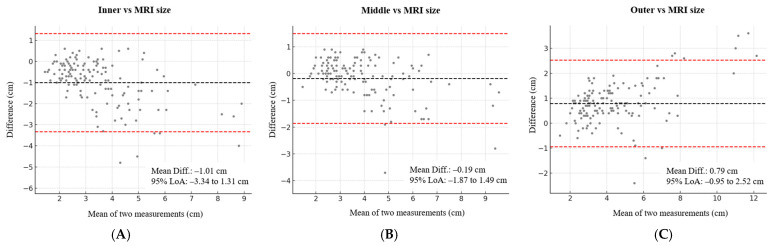
Bland–Altman plots comparing MRI and AI-CAD-guided tumor size based on (**A**) inner, (**B**) middle, and (**C**) outer contours. The middle contour showed the smallest mean difference (–0.19 cm; 95% limits of agreement –1.87 to 1.49 cm). AI-CAD = artificial intelligence-based computer-aided detection.

Bland–Altman analysis supported these findings (Figure 4). The mean difference ranged from –1.01 to 0.79 cm across all contours. The middle contour showed the smallest mean difference relative to MRI across lesion types: –0.19 cm (LoA, –1.87 to 1.49) in total cases, 0.07 cm (LoA, –0.92 to 1.07) in mass-only lesions, and –0.57 cm (LoA, –2.73 to 1.58) in NME-involved lesions. Across all contours, NME-involved lesions showed consistently greater mean differences than mass-only lesions, with mean differences ranging from –1.71 to 1.07 cm.

Concordance analysis ranged from 31.9% to 61.1%, with the highest rate observed for the middle contour (61.1%, 88/144). In mass-only lesions, the concordance rates were 70.9% (61/86) for the middle contour, 54.7% (47/86) for the inner contour, and 40.7% (35/86) for the outer contour. In NME-involved lesions, the middle contour also showed the highest concordance rate at 46.6% (27/58), while the inner and outer contours both showed identical rates of 19.0% (11/58 each).

Overall, the middle contour demonstrated the most consistent agreement with MRI tumor size measurements. Based on these results, the middle contour was used for AI-CAD-guided mammographic T stage comparisons in the following Section 3.4.

### 3.4. Concordance Between MRI and AI-CAD-Guided Mammographic T Staging

**Figure 5:**
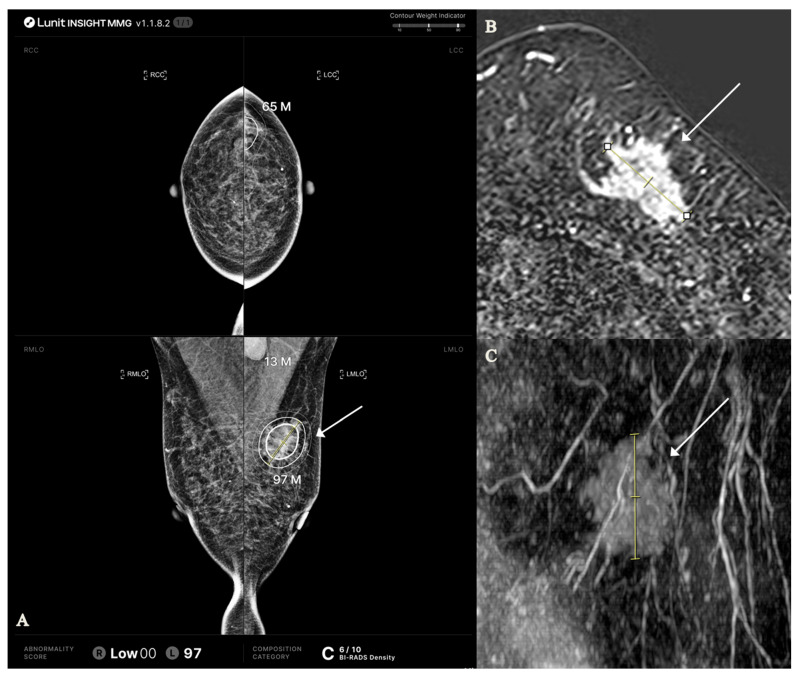
AI-CAD mammography and MRI findings in a 53-year-old woman with invasive ductal carcinoma, grade III (HR+/HER2−, Ki-67 index 85%). (**A**) AI-CAD mammography shows heterogeneously dense breasts (category C, density score 6/10) and a high abnormality score (97) on the left mediolateral oblique view. The lesion was classified as a mass. Tumor size along inner, middle, and outer contours was 2.6, 3.8, and 4.6 cm, respectively (T2 stage for all). A partially visualized enlarged axillary lymph node was also noted. (**B**,**C**) Contrast-enhanced MRI reveals an irregularly enhancing mass in the corresponding area. The lesion measured 2.8 cm on the axial view and 3.8 cm on the maximum intensity projection image; the maximal diameter (3.8 cm) was used for T2 staging. Arrows indicate the lesion; yellow lines represent caliper-based measurements. The outer contour in A was omitted due to visual overlap with other lines. AI-CAD = artificial intelligence-based computer-aided detection; HR = hormone receptor; HER2 = human epidermal growth factor receptor 2; MIP = maximum intensity projection.

As shown in Table 4, the confusion matrix for all cases demonstrated a quadratic weighted κ of 0.743 [95% CI, 0.634–0.852], indicating substantial agreement between MRI and AI-CAD-guided mammographic T staging (middle contour), with an overall agreement rate of 88.2% (127 of 144 cases) (Figure 5). Subgroup analysis showed higher concordance in mass-only lesions (κ = 0.725 [95% CI, 0.579–0.871]; agreement, 93.0%) compared to NME-involved lesions (κ = 0.624 [95% CI, 0.423–0.825]; agreement, 81.0%), with understaging occurring more frequently in the NME-involved group (17.2% vs. 2.3%). Confusion matrices for each subgroup are presented in Supplementary Table S1.

To further explore the concordance across clinicopathologic subgroups, a molecular subtype-stratified analysis was performed (Table 5). The highest concordance was observed in TNBC cases, with a κ of 0.902 [95% CI, 0.749–1.000] and an agreement rate of 95.4%, indicating almost perfect agreement. Other subtypes showed κ values ranging from 0.629 [95% CI, 0.316–0.854] to 0.704 [95% CI, 0.448–0.892], with agreement rates between 82.9% and 86.4%.

## 4. Discussion

In this study, we evaluated the concordance between AI-CAD-guided mammographic measurements and MRI-based assessments of tumor size and clinical T stage in 144 patients with breast cancer. Among the three AI-CAD-generated contours, the middle contour consistently demonstrated the highest agreement with MRI across all evaluation metrics. It showed good absolute agreement (ICC = 0.866), the smallest mean difference (–0.19 cm), and the highest concordance rate (61.1%). Based on these findings, the middle contour was selected as the representative contour for T stage comparison with MRI, showing substantial agreement (κ = 0.743; agreement, 88.2%).

The diagnostic performance of radiologists in mammographic interpretation can vary considerably [32,33,34]. To support consistent image evaluation, CAD systems were introduced, with the first approved by the U.S. Food and Drug Administration (FDA) in 1998 [35]. However, the effectiveness of conventional CAD has remained controversial due to low specificity, frequent false-positive markings, and high recall rates [36,37,38,39]. More recently, deep learning and CNNs have enabled substantial improvements in AI-based mammographic interpretation [40,41].

Lee et al. demonstrated that abnormality scores generated by the AI-CAD algorithm correlated well with PPVs, meeting BI-RADS recommendations [21]. Additionally, prior studies have shown that when radiologists could not identify corresponding imaging findings for AI-CAD detections, the likelihood of malignancy was extremely low [21,42]. Nonetheless, some argue that the abnormality score—ranging from 0 to 100—reflects only the degree of suspicion rather than a true probability of cancer [43].

While AI-CAD systems now offer lesion contours to assist with size estimation, their validity for tumor size measurement and T staging remains underexplored. To our knowledge, no prior studies have directly compared AI-CAD-generated measurements with established imaging standards. In this study, we assessed the agreement between AI-CAD-guided mammographic measurements and MRI tumor size determined by experienced radiologists.

Our Pearson correlation analysis demonstrated a linear relationship between tumor sizes measured by AI and those measured by MRI, although this did not imply perfect agreement in absolute values. While the middle and outer contours showed similar ICCs overall, the middle contour demonstrated a particularly high ICC of 0.883 in mass-only lesions. It also showed the smallest mean difference from MRI measurements across lesion types: –0.19 cm (LoA, –1.87 to 1.49) in total cases and 0.07 cm (LoA, –0.92 to 1.07) in mass-only lesions. These findings align with a previous study evaluating dual-layer CT (DLCT) with virtual monochromatic imaging (VMI), which reported an ICC of 0.840 and a mean difference of –0.05 cm compared to MRI (LoA, –1.29 to 1.19) [29].

A recent study using a Res-UNet model demonstrated high concordance between AI-generated and radiologist-derived MRI segmentations in mass lesions, with a T staging accuracy of 93% [44]. In contrast, our study focused on AI-CAD-guided mammographic measurements derived from contour levels, and showed substantial agreement with MRI-based T staging (κ = 0.743; agreement, 88.2%), particularly in mass-only lesions (κ = 0.725; agreement, 93.0%). Although our AI-CAD system was not designed to assign T stage directly, the radiologist-assigned staging based on AI-guided size showed promising concordance, suggesting potential utility in pre-treatment assessment.

Our subgroup analysis by molecular subtype revealed that HR–/HER2– (TNBC) cases showed a 95.4% agreement rate, likely due to their tendency to present themselves as mass lesions on MRI [45]. In contrast, NME-involved cases showed an 81.0% agreement rate and a 17.2% understaging rate. Given that HER2-positive cancers frequently present with NME, our findings should be interpreted with caution in this subgroup [46].

The BREAST-MRI randomized trial showed that preoperative MRI did not significantly improve local relapse-free or overall survival, nor did it reduce the reoperation rate [47]. In this context, our findings—demonstrating good concordance between AI-CAD-guided mammographic T staging and MRI—highlight the potential clinical utility of AI-assisted evaluation, particularly in settings where MRI is unavailable or impractical. Given that mammography and ultrasound are more widely accessible, AI-enhanced interpretation of conventional imaging may support appropriate treatment planning in resource-limited environments. In particular, AI-CAD-guided mammographic assessment may be clinically valuable in settings where MRI is not readily available due to cost, examination time, or limited imaging infrastructure, such as in community hospitals or rural clinics. In these contexts, AI-CAD can assist in identifying patients at higher risk of malignancy, thereby helping to triage cases and prioritize the use of advanced imaging modalities such as MRI for those with greater clinical need. Additionally, in situations where MRI is contraindicated—for example, patients with renal impairment, severe claustrophobia, or non-MRI-compatible implants—AI-CAD may provide tumor size estimates and diagnostic support.

Beyond detection and tumor sizing, AI is also being explored for risk stratification in breast cancer by analyzing mammographic features like breast density, with improved prediction over traditional models [16,48]. This expanding role of AI underscores its potential not only in detection but also in future clinical decision-making.

Nonetheless, our study has several limitations. First, this study was conducted at a single institution without the inclusion of a large, multi-center cohort. Although the internal consistency of our findings supports the validity of AI-CAD-guided lesion size assessment within this setting, the generalizability of the results to broader clinical environments may be limited due to potential variations in imaging protocols and patient characteristics across institutions. Second, our study included only patients who received NAC, and therefore those with ductal carcinoma in situ (DCIS) were not represented. As NAC is not indicated for DCIS, such patients were inherently excluded from the study cohort, which reflects current clinical practice. However, as AI-CAD performance may differ in preinvasive lesions or in patients not receiving NAC, caution is warranted in extrapolating our findings to such populations. Further prospective, multi-center studies involving more diverse patient cohorts are warranted to validate the broader applicability of AI-CAD in clinical staging. Third, we used only one commercially available AI system. Therefore, the applicability of our findings to other platforms with different algorithms remains uncertain. Fourth, a fixed AI-CAD abnormality score threshold (≥10) was applied as part of our inclusion criteria, based on the classification rule set by the Lunit INSIGHT MMG system. Scores below 10 are interpreted as negative, and no lesion contours are generated. The abnormality score represents the probability of malignancy generated by an algorithm-specific AI model and is influenced by the distribution and characteristics of its training data. Although the rationale for this cutoff is not publicly disclosed, it has been consistently used in prior studies employing the same software [21,22,42]. Finally, since our study included only patients who subsequently received NAC, pathological tumor size—altered by treatment-induced changes—could not be used as the reference standard. MRI, performed before NAC, is widely recognized as the most accurate imaging modality for evaluating tumor extent and, in patients receiving NAC, for assessing treatment response [9,10,11]. Accordingly, we considered MRI a reasonable and clinically relevant reference for evaluating the concordance of AI-CAD-guided mammographic tumor size estimation and clinical T staging. However, the absence of pathological confirmation limits the ability to assess how accurately AI-CAD reflects tumor extent, and this should be considered when interpreting the findings.

## 5. Conclusions

AI-CAD-guided mammographic tumor assessment, particularly when using the middle contour, demonstrated good agreement with MRI in estimating tumor size and clinical T stage. Given the limited accessibility of MRI in many clinical settings, this study was motivated by the need to evaluate the potential utility of AI-CAD-enhanced mammography as a supportive tool in clinical staging. These findings suggest that AI-CAD-enhanced mammography provides supportive information for clinical staging in breast cancer patients being considered for NAC, especially in settings where MRI is unavailable.

## Figures and Tables

**Table 1:** Baseline clinicopathologic and MRI characteristics of the study population.

Category	Variable	Value	*n* (%)
Demographics	Age (years)	Mean ± SD	51.7 ± 10.8
		<40 years	20 (13.9%)
		≥40 years	124 (86.1%)
	Menopausal state	Premenopause	76 (52.8%)
		Postmenopause	68 (47.2%)
	Family history of cancer	None	80 (55.6%)
		Breast cancer	20 (13.9%)
		Other cancer	44 (30.6%)
	BRCA1/2 mutation status	No	44 (30.6%)
		Yes	6 (4.2%)
		Unknown	94 (65.3%)
Pathology	Histologic type	Invasive ductal	134 (93.1%)
		Invasive lobular	5 (3.5%)
		Others	5 (3.5%)
	Histologic grade	I–II	80 (55.6%)
		III	63 (43.8%)
		Unknown	1 (0.7%)
	Estrogen receptor	No	63 (43.8%)
		Yes	81 (56.2%)
	Progesterone receptor	No	87 (60.4%)
		Yes	57 (39.6%)
	HER2 receptor	No	79 (54.9%)
		Yes	65 (45.1%)
	Molecular subtype	HR+/HER2–	35 (24.3%)
		HR+/HER2+	22 (15.3%)
		HR–/HER2+	43 (29.9%)
		HR–/HER2– (TNBC)	44 (30.6%)
	Ki-67 expression	<20%	23 (16.0%)
		≥20%	121 (84.0%)
MRI Characteristics	MRI BPE	Minimal	58 (40.3%)
		Mild	51 (35.4%)
		Moderate	19 (13.2%)
		Marked	16 (11.1%)
	MRI lesion type	Mass only	86 (59.7%)
		NME only	14 (9.7%)
		Mass with NME	44 (30.6%)
	MRI tumor size (cm)	Mean ± SD (range)	4.0 ± 1.9 (1.7–10.8)
	MRI-based T stage	T1	9 (6.2%)
		T2	100 (69.4%)
		T3	35 (24.3%)

MRI = magnetic resonance imaging; SD = standard deviation; HER2 = human epidermal growth factor receptor 2; HR = hormone receptor; TNBC = triple-negative breast cancer; BPE = background parenchymal enhancement; NME = non-mass enhancement.

**Table 2:** AI-CAD-guided mammographic lesion assessment.

Category	Variable	Value	*n* (%)
Breast Density	Breast composition	Fatty	1 (0.7%)
		Scattered	26 (18.1%)
		Heterogeneously dense	95 (66.0%)
		Extremely dense	22 (15.3%)
	Breast density score (0–10)	Mean ± SD (range)	6.9 ± 1.6 (3–10)
AI Abnormality Detection	Abnormality score (0–100)	Mean ± SD (range)	86.3 ± 22.2 (10–99)
	Abnormality type	Mass	58 (40.3%)
		Calcification	23 (16.0%)
		Mass with calcification	63 (43.8%)
	Detection view	CC	58 (40.3%)
		MLO	86 (59.7%)
	Number of abnormality contours	1	15 (10.4%)
		2	31 (21.5%)
		3	98 (68.1%)
Contour-based Tumor Size (cm)	Inner size	Mean ± SD (range)	3.0 ± 1.2 (1.2–7.9)
	Middle size	Mean ± SD (range)	3.8 ± 1.5 (1.2–9.2)
	Outer size	Mean ± SD (range)	4.8 ± 2.2 (1.2–13.5)
AI-CAD-guided T stage	Inner T stage	T1	34 (23.6%)
		T2	100 (69.4%)
		T3	10 (6.9%)
	Middle T stage	T1	5 (3.5%)
		T2	115 (79.9%)
		T3	24 (16.7%)
	Outer T stage	T1	2 (1.4%)
		T2	95 (66.0%)
		T3	47 (32.6%)

AI-CAD = artificial intelligence-based computer-aided detection; SD = standard deviation; CC = craniocaudal; MLO = mediolateral oblique.

**Table 3:** ICC, Bland–Altman, and concordance analysis of tumor sizes by MRI lesion type and AI-CAD-guided contours.

MRI Lesion Type	AI-CAD-Guided	ICC (95% CI)	Mean Diff. (AI–MRI) (cm)	SD of Diff. (cm)	Lower LoA (cm)	Upper LoA (cm)	Within ± 0.5 cm, *n* (%)
Total	Inner	0.602 (0.153, 0.794)	−1.01	1.18	−3.34	1.31	58 (40.3%)
(*n* = 144)	Middle	0.866 (0.815, 0.902)	−0.19	0.85	−1.87	1.49	88 (61.1%)
	Outer	0.847 (0.446, 0.936)	0.79	0.88	−0.95	2.52	46 (31.9%)
Mass only	Inner	0.714 (0.246, 0.870)	−0.54	0.62	−1.77	0.68	47 (54.7%)
(*n* = 86)	Middle	0.883 (0.827, 0.922)	0.07	0.50	−0.91	1.06	61 (70.9%)
	Outer	0.775 (0.133, 0.916)	0.60	0.54	−0.45	1.65	35 (40.7%)
NME-involved *	Inner	0.443 (0.000, 0.729)	−1.71	1.45	−4.56	1.14	11 (19.0%)
(*n* = 58)	Middle	0.780 (0.580, 0.880)	−0.57	1.10	−2.73	1.58	27 (46.6%)
	Outer	0.783 (0.301, 0.911)	1.07	1.18	−1.25	3.38	11 (19.0%)

* NME-involved includes both NME-only lesions and masses with associated NME. ICC = intraclass correlation coefficient; MRI = magnetic resonance imaging; AI-CAD = artificial intelligence-based computer-aided detection; CI = confidence interval; Diff. = difference; SD = standard deviation; LoA = limits of agreement; NME = non-mass enhancement.

**Table 4:** Confusion matrix for all cases and concordance metrics between MRI and AI-CAD-guided T staging: overall and subgroup analysis.

	AI-Based T Stage (Middle Contour)							
MRI T stage	T1	T2	T3	Total	Metrics	Total (*n* = 144)	Mass only (*n* = 86)	NME-involved * (*n* = 58)
T1	5	4	0	9	Quadratic weighted κ (95% CI)	0.743 (0.634, 0.852)	0.725 (0.579, 0.871)	0.624 (0.423, 0.825)
T2	0	99	1	100	Agreement rate (*n*, %)	127 (88.2%)	80 (93.0%)	47 (81.0%)
T3	0	12	23	35	Understaging rate (*n*, %)	12 (8.3%)	2 (2.3%)	10 (17.2%)
Total	5	115	24	144	Overstaging rate (*n*, %)	5 (3.5%)	4 (4.7%)	1 (1.7%)

* NME-involved: includes both NME-only and mass with NME lesions. MRI = magnetic resonance imaging; AI-CAD = artificial intelligence-based computer-aided detection; NME = non-mass enhancement; CI = confidence interval.

**Table 5:** Concordance metrics by molecular subtype (*n* = 144).

Molecular Subtype	*n*	Quadratic Weighted κ (95% CI)	Agreement (*n*, %)	Understaging (*n*, %)	Overstaging (*n*, %)
HR+/HER2–	35	0.629 (0.316, 0.854)	29 (82.9%)	5 (14.3%)	1 (2.8%)
HR+/HER2+	22	0.660 (0.282, 0.942)	19 (86.4%)	2 (9.1%)	1 (4.5%)
HR–/HER2+	43	0.704 (0.448, 0.892)	37 (86.0%)	4 (9.3%)	2 (4.7%)
HR–/HER2– (TNBC)	44	0.902 (0.749, 1.000)	42 (95.4%)	1 (2.3%)	1 (2.3%)

HR = hormone receptor; HER2 = human epidermal growth factor receptor 2; TNBC = triple-negative breast cancer; CI = confidence interval.

## Data Availability

The datasets generated or analyzed during the current study are not publicly available but may be made available by the corresponding author upon reasonable request.

## Supplementary Materials

The following supporting information can be downloaded at: https://www.mdpi.com/article/10.3390/tomography11070072/s1, Table S1: Confusion matrices for mass-only (*n* = 86) and NME-involved (*n* = 58) subgroups*.

## Abbreviations

The following abbreviations are used in this manuscript:

NAC	Neoadjuvant chemotherapy
MRI	Magnetic resonance imaging
AI-CAD	Artificial intelligence-based computer-aided detection
