# Supplementary material for: AI-CAD-Guided Mammographic Assessment of Tumor Size and T Stage: Concordance with MRI for Clinical Staging in Breast Cancer Patients Considered for NAC
Source: Tomography. 2025 Jun 24;11(7):72. doi: 10.3390/tomography11070072 (PMC12299520; doi:10.3390/tomography11070072)
Supplement: Supplementary file 1 [file tomography-11-00072-s001.zip › tomography-3688133-supplementary.pdf]

Supplementary Table S1. Confusion Matrices for Mass-Only (n = 86) and NME-Involved (n = 58) Subgroups*

	**AI-CAD-guided T stage (Middle Contour)**			
**MRI T stage**	**T1**	**T2**	**T3**	**Total**
T1	5	4	0	9
T2	0	72	0	72
T3	0	2	3	8
Total	5	78	3	86

	AI-CAD-guided T stage (Middle Contour)			
MRI T stage	T1	T2	T3	Total
T1	0	0	0	0
T2	0	27	1	28
T3	0	10	20	30
Total	0	37	21	58

*NME-involved: includes both NME-only and mass with NME lesions. MRI = magnetic resonance imaging; AI-CAD = intelligence-based computer-aided detection; NME = non-mass enhancement
